# Disparity in access for people with disabilities to outpatient dental care services: a retrospective cohort study

**DOI:** 10.1186/s12903-023-02948-6

**Published:** 2023-04-14

**Authors:** Bo-Young Park, Han-A Cho, Hosung Shin

**Affiliations:** 1 Department of Dental Hygiene, Shinhan University, Uijeongbu-Si, Republic of Korea; 2 Department of Social and Humanity in Dentistry, Wonkwang University School of Dentistry, 460 Iksan Dearo, Iksan, 54538 North Jula Korea

**Keywords:** Dental expenses, Dental utilization, Disability, Healthcare inequality, Regular source of care

## Abstract

**Background:**

People with disabilities face difficulties in oral health management and gaining access to dental care. The availability of a regular source of dental care (RSDC) is an important factor that influences the access to health services and care management. The purpose of this study was to determine the effect of the availability of RSDC on the number of annual dental visits and dental expenses per visit among people with disabilities.

**Methods:**

Data of 7,896,251 patients with dental problems in South Korea were analyzed using the 2002–2018 National Health Insurance claims data. A generalized estimating equation was applied to analyze the repeated-measurement data, and the interaction effect between RSDC and the disability severity was evaluated.

**Results:**

The number of annual dental visits was higher among people with (2.62) than among those without (2.23) disabilities. Despite their increased dental needs, both annual dental visits and dental expenses per visit were low among older individuals (*p* < 0.001). The proportion and frequency of annual dental visits was lower among women than among men with disabilities. RSDC had differential effects on the severity of disability. Compared to people without disabilities, RSDC increased the number of annual dental visits (*p* = 0.067) and the dental expenses per visit (*p* < 0.05) among those with severe disabilities, but the effect on the number of annual dental visits was not significant among those with mild disabilities (*p* = 0.698).

**Conclusions:**

Our results suggest a need for a special dental care system for people with disabilities, to ensure an RSDC, particularly for women and for older people with disabilities.

## Introduction

People with disabilities have more difficulty accessing healthcare services than do those without disabilities, because of mobility restrictions, social discrimination, and lower income due to disability [1]. Maltais et al. [2] reported that people with intellectual disabilities used optometry, physiotherapy, and Pap tests significantly less often than did people without disabilities. Rouleau et al. [3] also reported that 16.6% of the patients experienced difficulties in receiving dental treatment after they became disabled. In addition, previous studies on the oral health status of disabled people found that the prevalence of edentulous tooth loss and dental caries was higher among people with disabilities than in those without disabilities [4, 5]. Furthermore, the more severely disabled the patient, the greater the number of missing teeth and the lower the number of restored teeth [6]. The primary purpose of dental care use among people with disabilities was related to pain management, rather than oral disease prevention or regular checkups [7]. Income, education level, place of residence, demographic characteristics of caregivers, type of disability, and severity of disability affected the use of dental care by people with disabilities [3, 8].

A regular source of dental care (RSDC) is a factor that influences an individual's use of health services [9]. RSDC are related to receiving oral health services and have a positive effect on the oral health management of not only themselves but also their children [10, 11]. According to previous studies, there was a difference in having RSDC according to social disadvantages, such as income, education, occupation, medical insurance, age, marital status, and subjective health status [12–15]. However, despite many studies reporting a positive relationship between RSDC and medical use, there are few studies examining the effect of RSDC on healthcare use of people with disabilities. In addition, research focusing on RSDC was conducted before 2010 in dentistry, but recent research often approaches it in terms of regular visits.

The findings of previous studies on dental care use of disabled people were slightly different. A Korean study reported that disabled people use dental care 0.97 times less than non-disabled people [16]. However, other studies found that the dental service use rates of non-disabled and disabled people were similar or that people with disabilities used more dental services [17, 18]. To date, no studies have reported whether RSDC affects the frequency or cost of dental care over a long period. This study aimed to investigate the effect of RSDC on the use of dental care by people with disabilities using repeated measured claim data.

## Methods

### Research materials and participants

This retrospective study was approved by the Institutional Review Board of the Ethics and Scientific Review Committee of Wonkwang University (WKIRB-201911-SB-082) and performed in accordance with the Declaration of Helsinki. The Ethics committee waived the requirement of informed consent due to the retrospective study design and anonymity of the NHI claims data. Data management was conducted after receiving approval to use the database of the NHI Service and performed using a computer installed at a location with restricted external access The NHS DBs are relational databases, and variables were extracted by merging three DBs using the primary key; DB containing the information of the insured, the DB summarizing the treatments, and the DB containing the detailed treatment details. Data cleaning and management were performed by the authors and organized using the R 4.03 version (R Foundation for Statistical Computing, Vienna, Austria).

This study used cohort data (2002–2018) constructed from claims data of the Korean National Health Insurance (NHI). The NHI claims databases used in this study included data on healthcare use, detailed treatments, and sociodemographic information of the individuals. Sociodemographic data contained information on age, sex, region, income-based premiums, insurance type, and the type and grade of disability [19]. For analysis, the NHI data were organized in a cohort format. For example, NHI dental care users in 2002 were followed up until 2018 in a cohort format. Similarly, new dental users who did not overlap with previous years were added and were followed up to 2018. Overall, this study included 7,896,251 dental care users.

The dependent variables were the number of annual dental care visits and expenditure per visit. Dental expenses refer to the total expenditures, including the insurer’s contribution and the insured’s out-of-pocket expenses.

The Korean Welfare Act for Persons with Disabilities classifies persons with disabilities into 17 categories, and the severity of their disability is organized into six levels. For independent variables, grades 1, 2, and 3 were classified as severe disability, whereas grades 4, 5, and 6 were categorized as mild disability. A regular source of dental care was defined as continuous dental use for at least 2 years or more [20]. The age groups were categorized into children under 20 years, adults aged 20–64 years, and older individuals aged 65 years or older. Income levels were estimated based on the NHI premium (high, middle, and low), and residential areas were categorized into large cities, small cities, and rural areas. In addition, other variables, including medical aid and sex, were used as independent variables.

### Statistical analysis

Descriptive statistics were performed on the number of annual dental visits and dental expenses per visit using the chi-square test and one-way analysis of variance. The Scheffe post-hoc test was performed for multiple pairwise comparisons. The association between the dependent variable (the number of annual dental visits and dental expenses per visit) and availability of a regular source of care in each disability group were analyzed using generalized estimating equations (GEEs). GEEs are commonly used for analysis that considers intra-individual correlation due to repeated measures, not only in medicine and life sciences [21], but also in dentistry [22]. Notably, the GEE method calculates the population-averaged estimation on the premise that the independence assumption is violated because of the existence of a correlation between the residuals in linear regression analysis [23]. In our GEE analysis, male individuals, aged ≥ 65 years, living in large cities, with no regular source of dental care, non-medical aid, a low income, without disabilities were used as the reference group for analysis. Additionally, interaction effects were considered to determine the difference between the number of annual dental visits and dental expenses per visit based on a regular source of dental care and the severity of the disability. All analyses were performed using the R 4.03 version (R Foundation for Statistical Computing, Vienna, Austria) [24].

## Results

### Sample characteristics

The number of annual dental visits were in the following order: severely disability, mild disability, and no disability, whereas the dental expenses per visit were in the order of mild disability, no disability, and severe disability. Among people with disabilities, the proportion of men using dental services was approximately twice as high as that of females. More people with disabilities had an RSDC than did those with no disability. In the no-disability group, those with an RSDC was approximately 22% of those without a regular source of dental care, whereas the ratio was approximately 28% in the people with disabilities group. The proportion of medical aid users was highest among people with severe disabilities, which was approximately 13 times higher than that among people with no disability, and three times higher than that among people with mild disability. The income level distribution was similar to the medical aid distribution, and the lowest income group accounted for the largest portion of the people with severe disability group (Table 1).

**Table 1:** Sample characteristics

Variables	categories	Total (*N* = 7,896,251)	Severity of Disability			
			No disabilities (*N* = 7,514,703)	Mild disabilities (*N* = 232,062)	Severe disabilities (*N* = 149,486)	χ^2^ (P)^a^
		%	%	%	%	
Total		100	95.2	2.9	1.9	
Sex	Male	49.8	49.1	65.0	64.2	< 0.001
	Female	50.1	50.9	35.0	35.8	
Age	< 20	15.4	16.2	0.4	4.1	< 0.001
	20–64	74.7	74.6	79.8	77.4	
	≥ 65	9.7	9.3	19.8	18.5	
Residential areas	Large cities	60.1	60.5	54.1	53.6	< 0.001
	Rural areas	11.8	11.6	15.5	15.5	
	Small cities	27.9	27.9	30.4	30.9	
A regular source of dental care	No	81.6	81.9	79.2	76.6	< 0.001
	Yes	18.3	18.1	20.8	23.4	
Medical Aid	No	97.8	98.4	93.1	78.9	< 0.001
	Yes	2.1	1.6	6.9	21.1	
Income	Low	16.2	15.6	25.0	35.3	< 0.001
	Middle	58	58.3	56.3	48.4	
	High	25.7	26.1	18.8	16.3	
		Mean ± SD				*p*-value
Number of annual dental visits^‡^		2.25 ± 1.82	2.23 ± 1.79^a^	2.61 ± 2.11^b^	2.63 ± 2.64^c^	< 0.001
Dental expenses per visit^‡, §^		34.1 ± 22.7	34.1 ± 22.4^b^	36.2 ± 29.0^c^	32.2 ± 26.0^a^	0.000

^†^Chi-squared test

^‡^Analysis of variance (a < b < c)

^§^1000 KRW

### Number of annual dental visits and expenses per visit

The average annual number of dental visits was higher among males with disability and tended to decrease with increasing age in all three groups. An RSDC had a greater impact on dental use than did other variables, such as sex and age. Participants with an RSDC had 1.64 times more dental visits than did those without an RSDC. This trend was similar for both people with and those without disabilities. For example, in the absence of an RSDC, the average number of annual dental visits of people with severe disability was 2.27, which was about 15% higher than that among those without disabilities, but increased by 1.73 times when an RSDC was available.

**Fig. 1:**
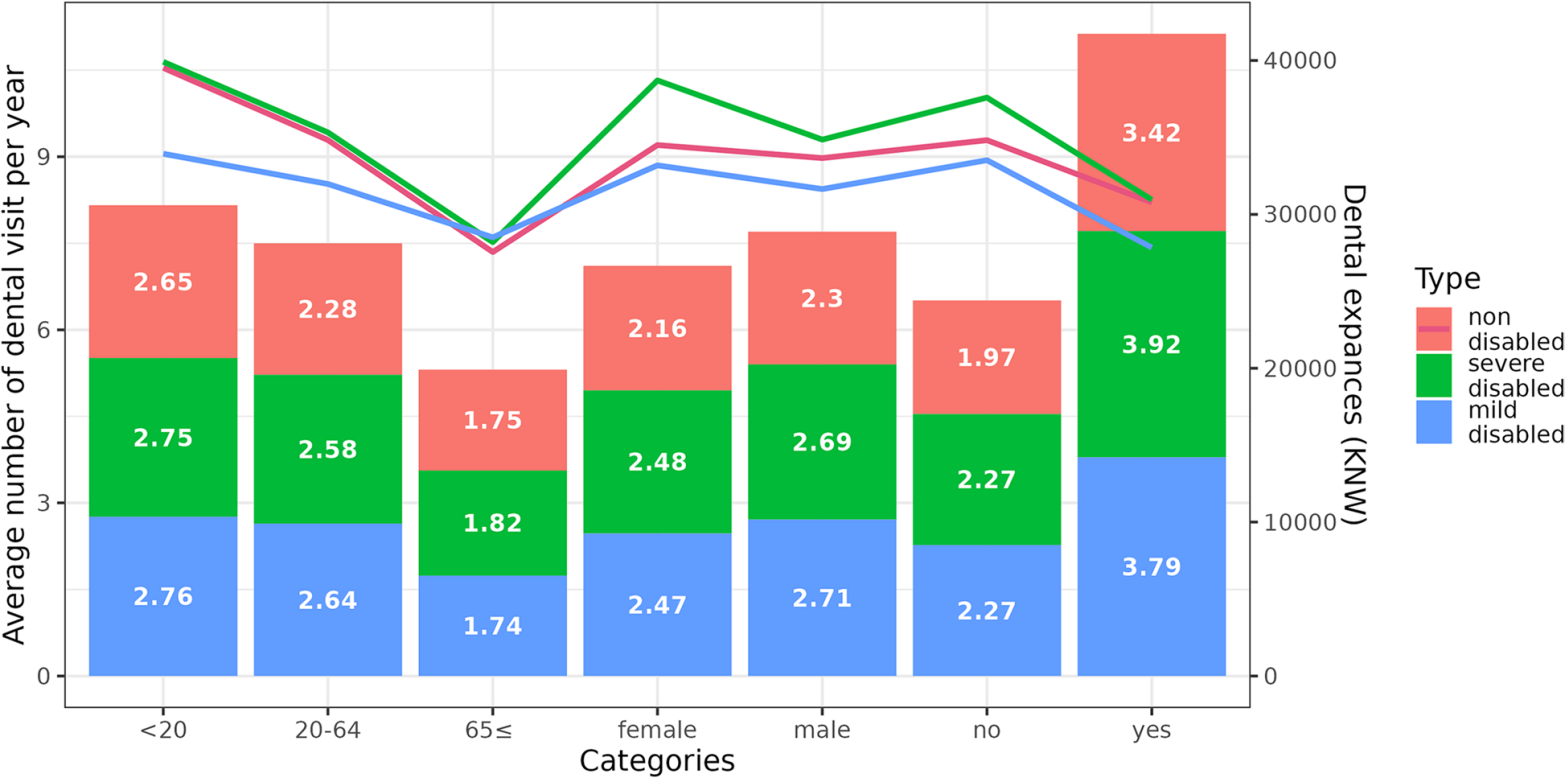
Annual number of dental visits and dental expenses for people with disabilities. Women with disabilities had fewer dental care visits than men with disabilities; however, they spent more on dental care per visit. Women with severe disabilities had the highest dental expenses per visit

Women had fewer dental visits than men; however, they spent more on dental care per visit. Women with severe disabilities had the highest dental expenses per visit (Fig. 1). As age increased, dental expenses per visit tended to decrease, and the number of dental visits in the group without an RSDC was low. However, group without a RSDC dental care expenses per visit were higher than those of their counterparts.

### Effect of a regular source of dental care on the annual number of dental visits and expenses

According to the GEE analysis, there was no difference in the average dental care use between men and women regardless of the statistical significance (the annual number of dental visits in males was only 4% higher than that in females.) Individuals 65 years and older had the lowest dental care use, whereas the group under 20 years old and the 20–64-year-old group had 51% and 26% higher dental care use, respectively, than the older group. In the no-disability group, the simple effect of an RSDC was 13.2%. In other words, when individuals had an RSDC, they would have more than a quarter (= 0.132*2.25 [average dental visits]) of the number of dental visits than those without an RSDC. The income and medical aid trends were similar. The higher the income level, the lower was the annual dental care use of individuals with no medical aid (Table 2).

**Table 2:** Factors affecting the annual number of dental care visits by people with disabilities

Variables	Category	Coefficient (coef)	Exp (coef)	95% CI		*p* value
				Lower	Upper	
Sex (Ref: Female)	Male	0.041	1.040	0.032	0.049	< 0.001
Age (Ref: ≥ 65 years)	< 20 years	0.414	1.510	0.401	0.426	< 0.001
	20–64 years	0.233	1.262	0.221	0.245	< 0.001
Residential areas (Ref: Large cities)	Small cities	0.020	1.010	0.004	0.033	0.004
	Rural areas	-0.020	0.980	-0.035	-0.006	0.007
RSDC ^a^ (Ref: No)	Yes	0.124	1.132	0.112	0.136	< 0.001
Medical aid (Ref: No)	Yes	0.054	1.056	0.028	0.079	< 0.001
Income (Ref: Low)	Middle	-0.009	0.991	-0.020	-0.002	0.104
	High	-0.014	0.986	-0.026-	-0.002	0.023
Disabilities (Ref: No disabilities)	Mild	0.059	1.061	0.040	0.078	< 0.001
	Severe	0.025	1.028	-0.006	0.056	0.121
Interaction Term RSDC ^a^: Disabilities	RSDC (Yes): Mild	-0.008	0.992	-0.050	0.0334	0.708
	RSDC (Yes): Severe	0.071	1.071	-0.001	0.144	0.055

^a^*RSDC *Regular source of dental care, *CI* Confidence interval, *coef* Coefficient

**Fig. 2:**
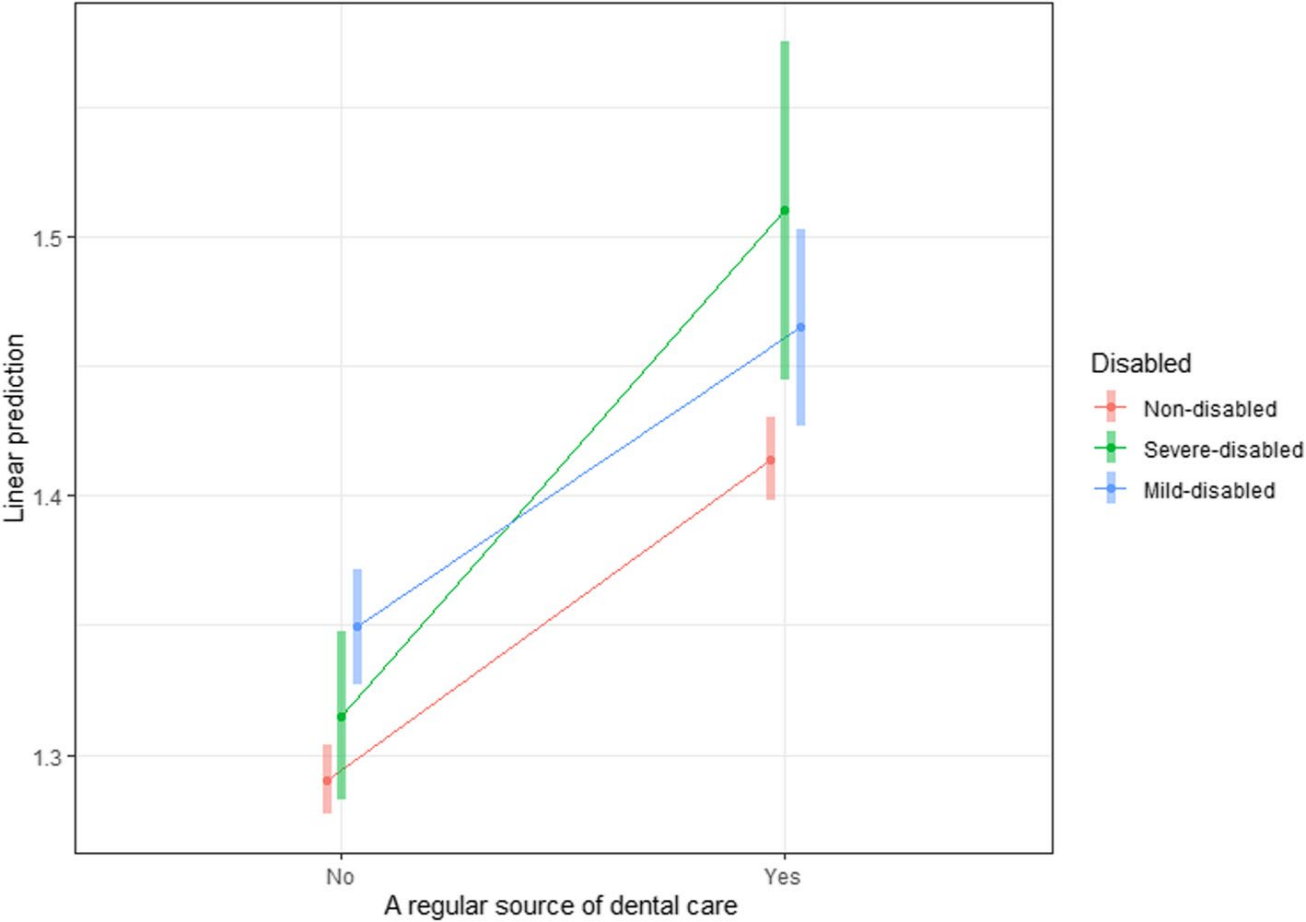
Interaction effect between a regular source of dental care and disability level. The effects of a regular source of dental care varied according to the disability severity. There was a marginally significant difference between individuals with severe disabilities and those with no disabilities.

The effects of an RSDC varied according to the severity of disability (Fig. 2). The interaction term for individuals with mild disability was not statistically significant; however, it was a marginally significant for those with severe disability. Considering the interaction effect between the severity of disability and availability of an RSDC, the simple effect of RSDC was approximately 21.5% (= exp [0.124 + 0.072]) for individuals with severe disability. This was greater than the simple effects among those with mild disability or with no disability, which were 12.3% and 13.2%, respectively.

In terms of dental expenses per visit (Table 3), older individuals spent the least, but those aged < 20 years spent more and had more dental visits than did those in other age groups. Individuals aged < 20 years tended to spend 95% more than did the older individuals.

**Table 3:** Factors affecting dental expenses per visit (generalized estimating equations)

Variables	Categories	Coefficient (coef)	Exp (coef)	95% CI		*p*-value
				Lower	Upper	
Sex (Ref: Female)	Male	-0.0005	1.000	-0.010	0.009	0.921
Age (Ref: ≥ 65 years)	< 20 years	0.667	1.948	0.654	0.679	< 0.001
	20–64 years	0.137	1.146	0.127	0.146	< 0.001
Residential areas (Ref: Large cities)	Small cities	-0.015	0.985	-0.032	0.003	0.103
	Rural areas	-0.003	0.997	-0.022	0.016	0.759
RSDC ^a^ (Ref: No)	Yes	-0.096	0.908	-0.109	-0.084	< 0.001
Medical aid (Ref: No)	Yes	0.105	1.110	0.068	0.141	< 0.001
Income (Ref: Low)	Middle	-0.005	0.995	-0.019	0.009	0.479
	High	0.052	1.054	0.037	0.068	< 0.001
Disabled (Ref: No disabilities)	Mild	0.063	1.065	0.036	0.091	< 0.001
	Severe	-0.006	0.994	-0.048	0.036	0.790
Interaction Term RSDC ^a^: Disabilities	RSDC (Yes): Mild	-0.082	0.921	-0.137	-0.027	0.003
	RSDC (Yes): Severe	-0.134	0.875	-0.222	-0.046	0.003

^a^ *RSDC *Regular source of dental care, *CI*: Confidence interval

**Fig. 3:**
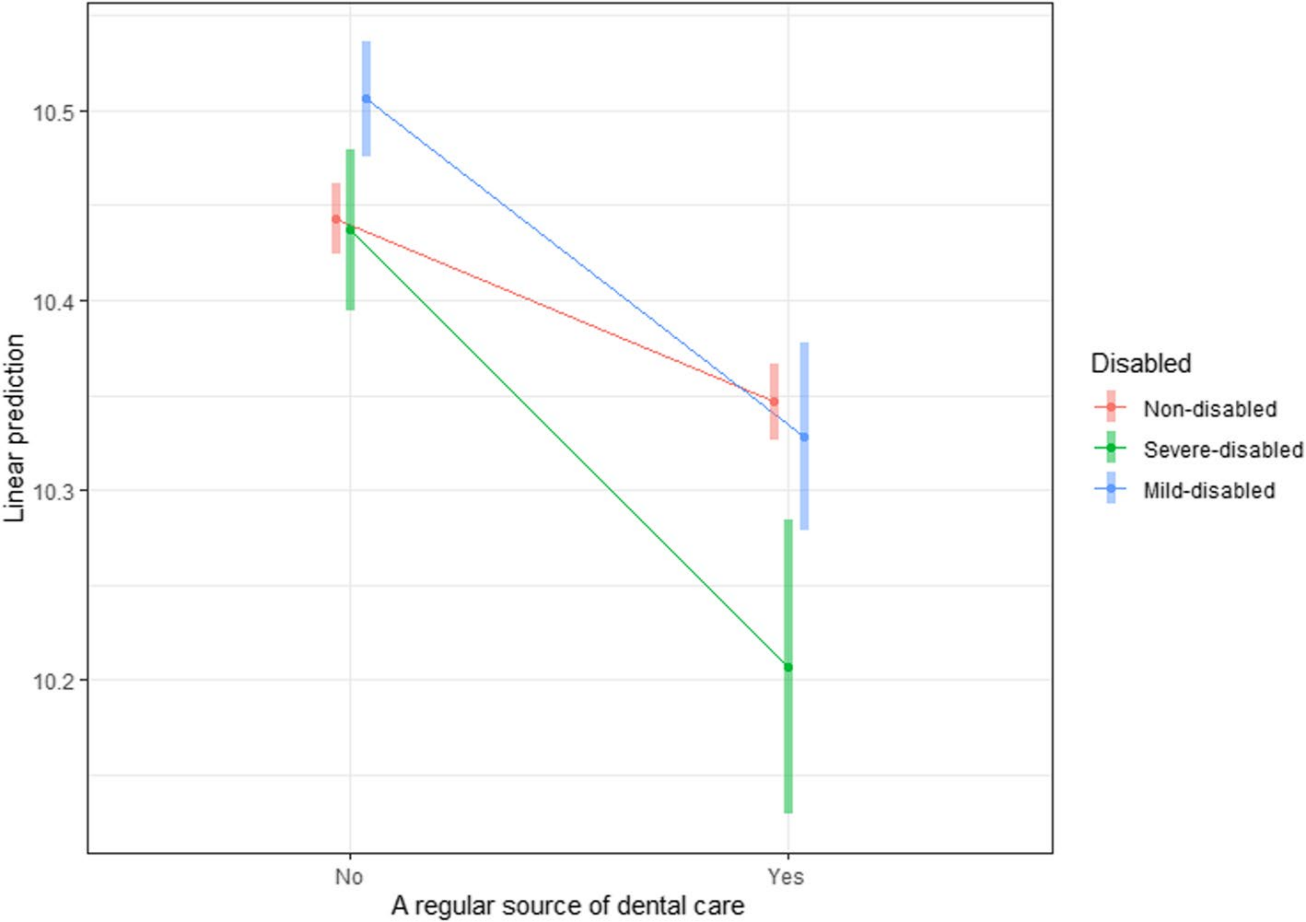
Interactions between the regular source of dental care and expenses per visit. The slope of the curve for people with mild and severe disabilities was steeper than that for people with no disabilities, implying that using a regular dental source of care would significantly reduce dental care costs per visit for people with disabilities.

Results using an interaction term are presented as a plot (Fig. 3) to examine whether the availability of an RSDC made a difference to the dental expenses per visit according to the severity of the disability. Each point shown in the plot is a predicted value. The slope of the line between the two points represents a simple effect. In Fig. 3, the slopes for those with mild and severe disability were significantly larger than that for those with no disability, implying that using an RSDC would significantly reduce the dental expenses per visit for people with disabilities.

## Discussion

This study analyzed the number of dental visits and expenses of people with disabilities using the 2002–2018 NHI claim data from South Korea and investigated their relationship with a regular source of care. We found that those with an RSDC visited dentists more often and that the effects of having an RSDC varied according to the severity of disability. Women and older individuals with disability used dental care less and spent more per dental visit than did their counterparts.

Physical barriers, care costs, and dental fear are common obstacles to dental care among people with disabilities [25]. People with disabilities had more decayed–missing–filled teeth, decayed teeth, and missing teeth than did those with no disabilities, resulting in poor oral health [4]. Lin et al. [26] reported that the dental service access rate was lower in people with disabilities than in the general population, but people with disabilities also had a higher dental filling rate, and periodontal treatment rate. A Brazilian study found that the proportion of disabled people using dental care was lower than that of people with no disability [27].

Park et al. [16] reported that the number of dental care users with disabilities was lower than that that of individuals with no disability, but the frequency was higher in those with disabilities than in those without disabilities. The annual number of dental visits in this study was 2.61 and 2.63 for people with mild disabilities and with severe disabilities, respectively, which was more than the 2.23 visits of people with no disability (Table 1). For people with disabilities, a retreatment visit might be necessary because the treated teeth could not be maintained in a healthy state due to the lack of preventive care [20] and difficulties in oral hygiene management. Furthermore, people with disabilities might need additional visits because of difficulty in giving cooperation during the dental visit [25]. In South Korea, access to most dental services, other than orthodontic and prosthodontic services, is less limited because the Korean NHI is available to all citizens, and out-of-pocket costs are lower for people with disabilities and for low income groups [22]. Based on the above-mentioned evidence, it was presumed that the frequency of dental care use of people with disabilities was higher than that of those with no disability.

The present study confirmed the inequality in dental care use between the sexes in people with disabilities. Among the NHI beneficiaries, the proportion of men with disabilities was approximately 10% higher than that of women with disabilities (data not presented), whereas the proportion of dental care users among men with disabilities was approximately twice that of women with disabilities. The average annual number of dental visits was higher among men with disabilities (Fig. 1). Gender inequality for people with disabilities has often been reported in terms of use of healthcare services [27]. In this study, women with disabilities visited dentists less frequently than did men with disabilities. Women with disabilities have been reported to have a lower level of education, a tendency to be poorer and to have lower employment prospects than men with disabilities [28]. The economic status of people with disabilities is related to the ability to pay for dental expenses and might act as one of major factors determining the number of dental visits as compared to that of individuals with no disability. A previous study [29] reported that approximately 60%–80% of people with disabilities had economic reasons for unmet dental needs. This proportion was higher than that of people without disabilities. In this study, people with disabilities with a high-income level had a higher annual number of dental visits. The difference in the annual number of dental visits according to the income level of people with disabilities was slightly larger than the difference in the no disability group. We speculate that women with disabilities might have a greater burden of dental visits due to paying for dental treatment than men with disabilities.

People with disabilities need a guardian or caregiver because of difficulties in mobility and appropriate communication when accessing dental care [25]. In South Korea, women with disabilities are reported to receive less obtaining care support than men with disabilities. A previous study [30] found that the average number of days of care per month was greater for men than for women with disabilities. In a study in the United States [31], women with disabilities received less home-care support than did men with disabilities. This suggests that gender inequality exists in the social support of people with disabilities.

Among people with disabilities, the average annual number of dental visits was decreased significantly in those aged 65 years and over, and the cost of dental treatments also tended to decrease by age groups (Fig. 1), which was consistent with the findings of previous study [32]. However, as age increases, the need for oral treatment tends to increase, due to chewing [33] and swallowing difficulties [34], and dry mouth [35], among other causes. In addition, activity restrictions [36] and economic factors [37] hinder the use of dental care. In a previous study, older people with restrictions in daily activities used dental care less often than those with restrictions in instrumental activities of daily living [36]. The oral health of older individuals is influenced by physical function and cognitive impairment [38, 39]. For older individuals in South Korea, health insurance benefits include dentures and implants services, as well as conventional oral treatments, including oral surgery [22]. Nonetheless, our study results showed that these Korean NHI benefit package was still not sufficient to reduce inequality in dental care among older people with disabilities.

In this study, an RSDC had a greater effect on dental treatment use than other factors, including sex and age. Considering the interaction effect between the severity of disability and RSDC in this study, the number of annual dental visits for people with severe disabilities with an RSDC was increased by 22%, i.e., it was 1.83 times higher than that of people without disabilities. However, the dental costs per visit of people with disabilities with an RSDC were reduced, and the effect was more marked in those with severe disabilities. Regular dental visits positively affects active oral health behaviors, including repeated dental visits, periodic preventive measures, and participation in oral health education [40, 41]. In a previous study, regular dental visits were beneficial for the early detection and treatment of oral diseases [42] and were associated with reducing the incidence of periodontitis [43]. A study of people aged ≥ 65 years reported that regular dental visits reduced the risk of severe disability [44]. In this regard, an RSDC will greatly contribute to regular dental visits of people with disabilities who are more vulnerable to poor oral health due to difficulties in communication and limited physical movement.

In terms of the availability of RSDC among people with disabilities, caregivers' awareness of the importance of RSDC, caregivers' active attitude toward oral health care, and policies that encourage dentists to actively perform dental treatment for people with disabilities seem to be necessary (e.g., dental care facility expansion, care incentives for providers, and improving awareness of health disparities among people with disabilities) [45, 46]. According to a previous study [47] that investigated the barriers to oral health among people with disabilities, 54% of dentists reported they would not treat people with cognitive impairment and a poor ability to collaborate during treatment, and 50% of dentists who treated people with cognitive impairment reported that they did not include such patients in follow-up. As the importance of regular dental care for people with disabilities has been established, the need for active use of teledentistry has been emphasized [48]. In particular, during the COVID-19 pandemic, teledentistry reduced the risk of cross-contamination, enabled regular examinations, and helped reduce the occurrence of emergencies [49.50]. Thus, teledentistry has been proposed as an efficient and effective way for people with mobility restrictions or social barriers, such as people with disabilities, to regularly evaluate their oral health without visiting the dentist [49, 50].

This study had some limitations. This study analyzed data of people who had used dental care services; thus, caution is needed in interpreting the results. In this study, the number of annual dental visits was higher in people with disabilities than in those with no disability, but the analysis was based only on people with disabilities who used dental care. Therefore, our finding does not reflect the difference in dental care use among the entire population. In future studies, it is necessary to examine the effects of an RSDC and inequality in dental care among all people with disabilities, including those who do not use dental care. Additionally, caregivers significantly affected the dental visits of people with disabilities; however, the study did not investigate caregivers’ factors because of the limitations of the NHI data. Nevertheless, this study was significant in that it analyzed the dental care use of people with disabilities using repeatedly measured, long-term Korean NHI data and confirmed the positive effect of an RSDC.

## Conclusion

This study showed that patients with an RSCD had more annual dental visits and lower dental expenses. For older individuals, despite the increased dental needs associated with age, the number of dental visits and associated expenses were low. A similar trend was also observed in women with disability. For those with severe disability, an RSDC was effective in increasing the number of dental visits and reducing dental expenses. Based on this study’s findings, policies that can help to provide an RSDC for people with disabilities and to resolve inequality in dental services for women and older individuals with disabilities would result in significant improvement in the use of dental care.

## Data Availability

The data that support the findings of this study are available from database of National Health Insurance https://nhiss.nhis.or.kr/ but restrictions apply to the availability of these data, which were used under license for the current study, and so are not publicly available. Data are, however, available from the corresponding authors upon reasonable request and with permission of Korean National Health Insurance.
